# Effectiveness of vortioxetine for major depressive disorder in real-world clinical practice: US cohort results from the global RELIEVE study

**DOI:** 10.3389/fpsyt.2022.977560

**Published:** 2023-01-04

**Authors:** Gregory Mattingly, Elizabeth Brunner, Lambros Chrones, Debra F. Lawrence, Kenneth Simonsen, Hongye Ren

**Affiliations:** ^1^St. Charles Psychiatric Associates, St. Charles, MO, United States; ^2^Lundbeck LLC, Deerfield, IL, United States; ^3^Takeda Pharmaceuticals U.S.A., Inc., Lexington, MA, United States; ^4^H. Lundbeck A/S, Valby, Denmark

**Keywords:** cognition, functioning, major depressive disorder, real-world evidence, vortioxetine

## Abstract

**Introduction:**

Effective treatment of major depressive disorder (MDD) involves addressing both depressive and functional symptoms, increasing patients’ overall sense of well-being and quality of life (QoL).

**Methods:**

RELIEVE was an international observational, prospective study in patients ≥18 years with a current diagnosis of a major depressive episode (MDE) initiating vortioxetine in routine clinical practice; outcomes for the cohort of participants from the United States are presented here. Functioning was assessed at weeks 12 and 24 versus baseline using the Sheehan Disability Scale (SDS). Secondary effectiveness analyses assessed changes from baseline to weeks 12 and 24 in functioning, depression severity, cognitive symptoms, sexual function, and QoL.

**Results:**

244 participants had an average of 8.2 previous MDEs; mean duration of their current MDE at baseline was 93.5 weeks. Vortioxetine was used as second- or later-line treatment for 80.5% of participants. Least-squares mean (SE) SDS total score significantly decreased from baseline by 7.19 (0.52) points at week 12 and 8.19 (0.56) points at week 24 (*p* < 0.0001 for both). Significant improvements were also reflected across SDS subscores, depression severity, cognitive function, sexual function, and QoL. Healthcare resource utilization and productivity parameters also improved. Adverse events were observed in 21.8% of patients, with nausea being the most common (7.3%).

**Conclusion:**

This real-world study demonstrated improvements in functioning, depressive symptoms, and cognitive function in patients with MDD treated with vortioxetine in routine clinical practice in the cohort of patients enrolled in the United States. Outcomes were consistent with the efficacy and safety profile of vortioxetine in randomized controlled trials.

## Introduction

Every year, an estimated 21 million adults in the United States have at least one major depressive episode (MDE), meaning that 8.4% of all US adults have major depressive disorder (MDD) (1). The societal impact and economic burden of MDD has also increased in the United States, with direct and indirect healthcare, suicide-related, and workplace costs increasing by almost 40% to $326.2 billion between 2010 and 2018 (2). In particular, the costs associated with absenteeism and presenteeism can be equivalent to >30% of an individual’s annual salary (3).

Optimal treatment needs to address both the depressive symptoms and overall functioning of the diverse array of patients with MDD while also minimizing the risk of adverse events (AEs) and increasing the patient’s overall sense of well-being and quality of life (QoL) (4) given that approximately two-thirds of adults with MDD have severe functional impairment prior to treatment (5). In addition, up to 94% of adults with MDD experience cognitive symptoms during an MDE (6), including impairments in executive function, attention, learning and memory, and processing speed (7, 8), which can contribute to MDD-related social, functional, and work-related disability (8). Therefore, patients place a high level of importance on achieving functional remission with treatment, as well as relieving depressive symptoms (4).

However, treatment in both clinical and research settings tends to focus on addressing the depressive symptoms of MDD, with lesser consideration given to functioning and cognitive symptoms (9). Therefore, while antidepressant therapies may be efficacious in relieving depressive symptoms, their real-world effectiveness may be limited if treatment fails to adequately address functioning and cognitive symptoms, which may also increase the risk of a recurrent MDE (9).

For example, patients with MDD in the United States are most commonly treated with selective serotonin reuptake inhibitors (SSRIs), but approximately half will require a second-line therapy, and one-fourth will require third-line therapy (10) because most antidepressant therapies, including SSRIs, do not improve cognitive symptoms and altered psychosocial functioning associated with MDD (6, 11, 12). Antidepressant therapy also can be associated with new or worsening treatment-emergent sexual dysfunction, which negatively affects patients’ QoL (13).

Vortioxetine, a multimodal oral antidepressant, was approved by the US Food and Drug Administration (FDA) for the treatment of MDD in 2013 (14). Randomized clinical trials of vortioxetine therapy for patients with MDD have demonstrated efficacy and safety, as well as improved overall and cognitive functioning (8, 15, 16). Vortioxetine also is associated with limited treatment-related sexual dysfunction (17, 18). The FDA continues to monitor the real-world usage, safety, and effectiveness of treatments in the United States post-approval and uses these real-world data to guide regulatory decisions (19). In particular, data generated from multinational clinical trials may not be readily generalizable to a US population given the unique epidemiologic profile of MDD in the United States, characterized by a high overall prevalence and earlier age of diagnosis (20). Patients in the United States are also more likely to be prescribed antidepressant therapy and receive lifestyle advice about diet, exercise, and alcohol consumption than patients in other countries (21).

The Real Life Effectiveness of Vortioxetine in Depression (RELIEVE) study aimed to evaluate the real-world effectiveness of vortioxetine on overall functioning, including the impact of vortioxetine treatment on cognitive function, depressive symptoms, patient QoL, and safety and tolerability in patients with MDD seen in routine clinical practice in the United States, Canada, France, and Italy (22). However, the effectiveness of psychopharmacologic therapies can vary between countries, with treatment potentially being less effective as national per capita income and healthcare expenditures increase (23). Therefore, this paper presents the study findings for the patient cohort enrolled in the United States, a country with a unique patient population with MDD (20, 21) and the highest healthcare expenditure per capita in the world (24).

## Materials and methods

### Study design

RELIEVE was an observational, prospective cohort study of men and women aged ≥18 years with a current diagnosis of an MDE who were initiating treatment with vortioxetine in routine clinical practice. The study design and eligibility criteria have been previously described in detail in the global RELIEVE study publication (22); this analysis reports data from the subgroup of participants enrolled at 36 general practice and psychiatric outpatient clinics in the United States.

The study was conducted in accordance with Good Clinical Practice guidelines and FDA regulations, with ethical approval provided by each participating site. The study was prospectively registered on ClinicalTrials.gov (NCT03555136). All participants provided written informed consent prior to enrollment.

### Study assessments and analyses

Participants were assessed during visits at baseline, week 12 (±4 weeks), and week 24 (±4 weeks). Baseline demographics and history of MDD were collected at baseline, while outcomes assessing the effectiveness of vortioxetine were performed at each subsequent study visit.

The primary effectiveness analysis assessed the change in functioning from baseline to weeks 12 and 24 as measured by the Sheehan Disability Scale (SDS), a validated tool for assessing functional impairment in patients with MDD. SDS scoring ranges from 0 to 30, with higher scores indicating greater impairment in functioning (12).

Secondary effectiveness analyses assessed the changes from baseline to weeks 12 and 24 in functioning, depression severity, cognitive symptoms, sexual function, and QoL. Functioning domains (work/school, social life, and family life/home responsibilities) were assessed using SDS subscores. Depression severity was assessed using the Patient Health Questionnaire–9 item (PHQ-9), Clinical Global Impression Scale–Severity (CGI-S), and Clinical Global Impression Scale–Improvement (CGI-I), with higher scores indicating more severe symptoms. The Digit Symbol Substitution Test (DSST) and Perceived Deficits Questionnaire–Depression–5 item (PDQ-D5) were used to investigate cognitive function and cognitive symptoms. Sexual function was assessed using the Arizona Sexual Experience Scale (ASEX), and the EuroQoL 5 Dimensions 5 Levels (EQ-5D-5L) utility index was used for QoL. Data on healthcare resource utilization and workplace effects, including absenteeism and presenteeism, were also collected. AEs were recorded using the Medical Dictionary for Regulatory Activities (MedDRA) version 23.1.

### Statistics

Analyses were performed on all participants enrolled in the United States who initiated vortioxetine treatment and completed the baseline visit and ≥1 follow-up assessment visit. Descriptive statistics are presented. The primary and secondary endpoints were assessed using a linear mixed model for repeated measures adjusted for clinically relevant covariates (i.e., age, sex, education level, duration of MDE at baseline, baseline comorbidities, and baseline depression severity [PHQ-9]). Analyses were performed with the statistical software R^®^, version 3.6.1.

## Results

### Patient disposition and baseline characteristics

In total, 381 participants in the United States were included in the safety analysis population and 246 were included in the effectiveness population. Mean participant age was 44 years, and the majority of patients were female (66.7%) and White (84.1%) (Table 1). Participants reported an average of 8.2 previous MDEs, with a mean duration of their current MDE at baseline of 93.5 weeks. For most participants, vortioxetine was used as a second- or later-line treatment (80.5%) with a starting dose ≤10 mg (86.6%; mean baseline dose [SD] US cohort: 9.25 [4.75] mg; global cohort: 9.58 [5.45] mg).

**TABLE 1:** Baseline characteristics and demographics of participants in the RELIEVE study United States analysis.

Characteristics	*N* = 246
Sex, female, *n* (%)	164 (66.7)
Age, years, mean (SD) >65 years, *n* (%)	44.0 (14.8) 22 (8.9)
**Race/ethnicity, *n* (%)** White Black/African American Hispanic/Latino Asian Unspecified	207 (84.1) 16 (6.5) 13 (5.3) 5 (2.0) 5 (2.0)
**BMI,^a^ kg/m^2^, mean (SD)**	29.9 (7.4)
**Comorbidities (≥5% of participants), *n* (%)** Overweight/obese (≥25.0 kg/m^2^) Anxiety Sleep disorder Cardiovascular disease Chronic pain Neurologic disorder Diabetes Chronic fatigue	191 (77.6) 179 (73.1) 161 (65.4) 48 (19.5) 31 (12.6) 29 (11.8) 19 (7.7) 14 (5.7) 13 (5.3)
**Occupation, *n* (%)** Working Non-working	157 (63.8) 89 (36.2)
**MDD history,^b^ mean (SD)** Number of previous MDEs Duration of current MDE, weeks	8.2 (13.6) 93.5 (227.1)
**Time (years) since MDD diagnosis, mean (SD)**	14.6 (11.6)
**Vortioxetine treatment line, *n* (%)** First Second Third +	48 (19.5) 91 (37.0) 107 (43.5)
**Outcome assessments at baseline, mean (SD)** SDS total score PHQ-9 CGI-S DSST PDQ-D5 ASEX EQ-5D-5L	19.6 (6.2) 16.0 (5.7) 4.3 (0.9) 51.3 (14.0) 12.2 (4.9) 19.0 (5.8) 0.7 (0.2)

^a^*n* = 245; ^b^*n* = 244.

ASEX, Arizona Sexual Experience Scale; BMI, body mass index; CGI-S, Clinical Global Impression Scale–Severity; DSST, Digit Symbol Substitution Test; EQ-5D-5L, EuroQoL 5 Dimensions 5 Levels; LS, least squares; MDD, major depressive disorder; MDE, major depressive episode; PDQ-D5, Perceived Deficits Questionnaire–Depression–5 item; PHQ-9, Patient Health Questionnaire–9 item; SDS, Sheehan Disability Scale.

Most participants had a body mass index that would place them in the overweight or obese category (73.1%), and approximately three-fourths had at least 1 comorbidity. Almost two-thirds (65.4%) of participants had comorbid anxiety, and 19.5% had sleep disorders.

At baseline, the mean PHQ-9 score was 16.0, indicating moderately severe depression. Mean SDS total score at baseline was 19.6, with most (89.8%) participants having moderate (SDS total score 12–20) or severe (SDS total score 20–30) functional impairment at baseline.

### Functional impairment

**FIGURE 1:**
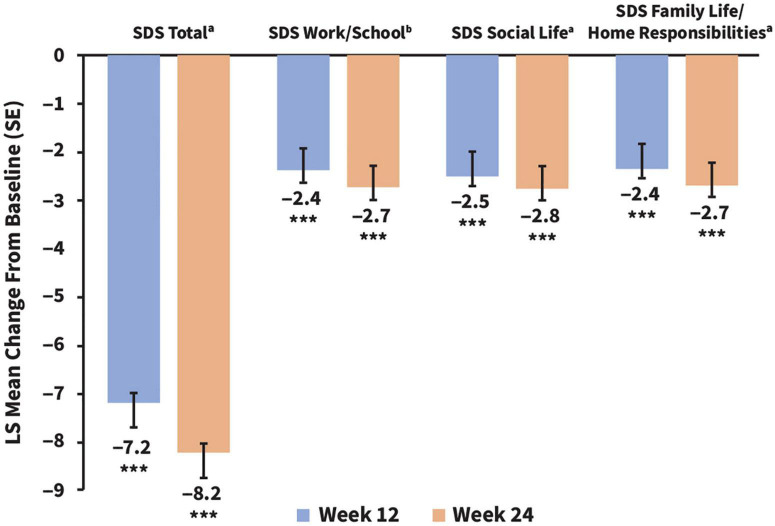
Changes in adjusted LS mean SDS total scores and subscores from baseline to weeks 12 and 24. ***p < 0.001. All statistical comparisons were significant vs. baseline. SDS LS mean baseline scores (CI) for comparison were as follows: total, 19.58 (18.78–20.38); work/school, 6.28 (5.91–6.64); social life, 6.72 (6.42–7.02); and family life/home responsibilities, 6.55 (6.26–6.84). ^a^*n* = 233 (week 12) and *n* = 203 (week 24), ^b^*n* = 196 (week 12) and *n* = 169 (week 24). LS, least squares; SDS, Sheehan Disability Scale.

After initiating vortioxetine therapy, the least-squares (LS) mean for the SDS total score improved from 19.49 (95% CI, 18.57–20.42) at baseline to 12.30 (95% CI, 11.10–13.50) at week 12 and 11.30 (95% CI, 10.09–12.51) at week 24. This corresponded to a significant change in LS mean (SE) SDS total score from baseline of –7.19 (0.52) points at week 12 and of –8.19 (0.56) points at week 24 (*p* < 0.0001 at both time points) (Figure 1). Similar improvements were reflected across all SDS subscores (*p* < 0.0001 for all) (Figure 1).

**FIGURE 2:**
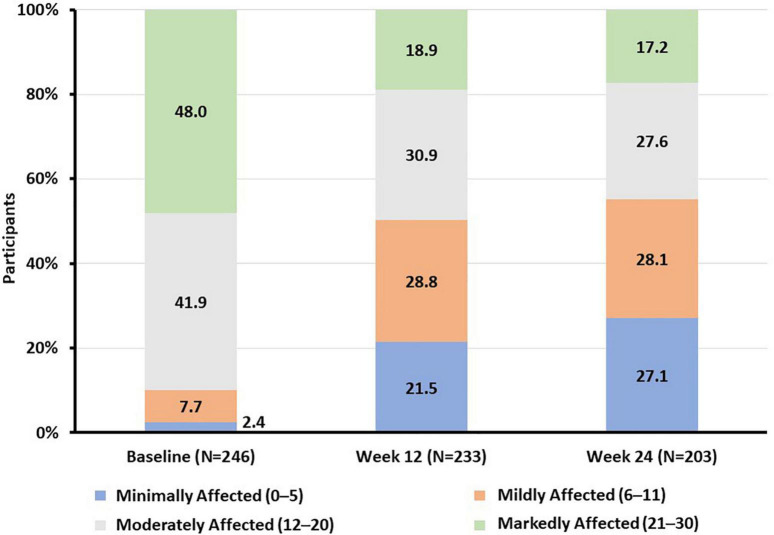
Proportion of participants according to SDS total score functional impairment category at baseline and weeks 12 and 24. SDS, Sheehan Disability Scale.

The proportion of participants with moderate to severe functional impairment decreased from 89.8 to 49.8% at week 12 and 44.8% at week 24 of vortioxetine treatment, respectively (Figure 2). After 24 weeks of treatment with vortioxetine, more than half (55.2%) of the study population reported mild or minimal functional impairment compared with only 10.2% of participants at study baseline (Figure 2).

### Depression severity

Significant improvements in depression severity were seen at weeks 12 and 24 (Table 2). Adjusted mean PHQ-9 score was reduced from 16.31 (95% CI, 15.34–17.29) at baseline to 10.67 (95% CI, 9.62–11.72) at week 12 and 9.63 (95% CI, 8.57–10.69) at week 24. Adjusted mean CGI-S score was reduced from 4.28 (95% CI, 4.16–4.41) at baseline to 3.23 (95% CI, 3.06–3.39) at week 12 and 2.92 (95% CI, 2.74–3.10) at week 24. Mean (SD) CGI-I was 2.7 (1.13) at week 12 and 2.4 (1.13) at week 24.

**TABLE 2:** Changes in adjusted LS mean scores from baseline to weeks 12 and 24 for depression severity, cognitive and sexual function, and QoL.

	Baseline score, adjusted LS mean (95% CI)	Change from baseline to week 12, adjusted LS mean (SE)	Change from baseline to week 24, adjusted LS mean (SE)
PHQ-9^a^	16.31(15.34–17.29)	−5.64(0.41)***	−6.68(0.46)***
CGI-S^b^	4.28(4.16–4.41)	−1.06(0.07)***	−1.37(0.09)***
CGI-I^b^	–	2.7 (0.07)	2.4 (0.07)
DSST^c^	47.05(44.82–49.27)	5.50(0.86)***	5.79(1.15)***
PDQ-D5^d^	11.62(10.91–12.33)	−3.47(0.32)***	−4.08(0.35)***
ASEX^d^	18.85(17.94–19.76)	−1.04(0.28)***	−1.12(0.35)**
EQ-5D-5L^d^	0.66(0.64–0.68)	0.08(0.01)***	0.09(0.01)***

No statistical analysis was performed for CGI-I because this tool does not include a numerical baseline value.

^a^*n* = 245 (baseline), *n* = 232 (week 12), and *n* = 203 (week 24); ^b^*n* = 246 (baseline), *n* = 235 (week 12), and *n* = 205 (week 24); ^c^*n* = 246 (baseline), *n* = 231 (week 12), and *n* = 194 (week 24); ^d^*n* = 246 (baseline), *n* = 233 (week 12), and *n* = 203 (week 24).

**p < 0.01; ***p < 0.001.

ASEX, Arizona Sexual Experience Scale; CGI-I, Clinical Global Impression Scale–Improvement; CGI-S, Clinical Global Impression Scale–Severity; CI, confidence interval; DSST, Digit Symbol Substitution Test; EQ-5D-5L, EuroQoL 5 Dimensions 5 Levels; LS, least squares; PDQ-D5, Perceived Deficits Questionnaire–Depression–5 item; PHQ-9, Patient Health Questionnaire–9 item; QoL, quality of life; SE, standard error.

### Cognitive function

Significant improvements from baseline were also seen across cognitive function and symptom parameters at weeks 12 and 24 (Table 2). Adjusted mean DSST score increased from 47.05 (95% CI, 44.82–49.27) at baseline to 52.55 (95% CI, 50.02–55.08) and 52.84 (95% CI, 49.87–55.80) at weeks 12 and 24, respectively. Adjusted mean PDQ-5 score also was improved over the treatment period from 11.62 (95% CI, 10.91–12.33) at baseline to 8.15 (95% CI, 7.32–8.98) at week 12 and 7.54 (95% CI, 6.70–8.37) at week 24.

### Sexual function

A sustained and statistically significant improvement in adjusted mean ASEX scores was reported, declining from 18.85 (95% CI, 17.94–19.76) at baseline to 17.81 (95% CI, 16.88–18.74) and 17.73 (95% CI, 16.79–18.67) at weeks 12 and 24, respectively (Table 2). At baseline, 97 patients (64.7%) reported sexual dysfunction compared with 77 (54.2%) and 79 (53.7%) patients after 12 and 24 weeks of vortioxetine treatment. Of the 51 patients without sexual dysfunction at baseline, 14 (27.5%) reported treatment-emergent sexual dysfunction at week 24.

### Quality of life

EQ-5D-5L utility index score also improved over the treatment period from 0.66 at baseline to 0.74 (95% CI, 0.71–0.76) at week 12 and 0.75 (95% CI, 0.73–0.77) at week 24 (Table 2).

### Healthcare resource utilization, absenteeism, and presenteeism

Mean (SD) total number of healthcare visits for any reason were reduced by 1.4 (4.87) per 3 months at week 12 and 1.9 (5.24) per 3 months at week 24 from a baseline of 4.6 (6.43) visits in the 12 weeks prior to initiating vortioxetine. The mean (SD) change in absenteeism versus baseline, defined as SDS days lost per week in the working population, was –0.8 (2.19) and −0.9 (2.21) at weeks 12 and 24, respectively. Likewise, there was a decreasing trend in presenteeism, derived from SDS based on underproductive days per week for the working population, at weeks 12 (–1.3 [2.90]) and 24 (–1.9 [2.62]) compared with baseline. A positive relationship was noted between change in depression severity and improvement in presenteeism at weeks 12 (correlation coefficient *r* = 0.23; 95% CI, 0.18–0.28) and 24 (*r* = 0.19; 95% CI, 0.13–0.25) (*p* < 0.0001 at both time points).

### Safety and tolerability

Vortioxetine was well tolerated in this real-world population with MDD in the United States and in line with its established safety profile. In total, 100 AEs were reported by 83 (21.8%) patients. The most frequently reported AEs were nausea (7.3%), pruritus (1.6%), and vomiting (1.3%). Discontinuation of the study drug due to lack of tolerability occurred in 15 participants at week 12 (6.1%) and 4 participants at week 24 (1.6%).

## Discussion

This study demonstrates a real-world improvement in functioning among the cohort of patients with MDD treated with vortioxetine in the United States. Improved cognitive function according to DSST and PDQ-5 scores, and depression severity according to PHQ-9 score, were also observed. The outcomes of this study are notable because the high mean number of prior MDEs (>8 per patient), long duration of current MDE (mean of approximately 1.8 years), and more than 80% of patients receiving prior treatment for MDD indicate that the patient cohort represents what could be considered a more difficult-to-treat patient population in routine clinical practice in the United States (25). The delay in effective treatment for these patients potentially results in a prolonged time to functional recovery and remission (4).

Patients in this US cohort differed from the overall RELIEVE Global cohort (22), as they were younger, more likely to be overweight/obese, had almost twice as many previous MDEs, double the mean duration of the current MDE, and were more likely to be receiving vortioxetine as a third or later line of therapy compared with >75% of patients in the global cohort who received vortioxetine as first- or second-line therapy. This is consistent with the vast majority (90%) of patients in the United States being prescribed an SSRI or a serotonin–norepinephrine reuptake inhibitor (SNRI) as antidepressant therapy (21), so switching to vortioxetine therapy may only have occurred after several prior therapies failed to adequately control MDD. The characteristics of the US cohort are also consistent with previous reports of patients with MDD being younger and more likely to be prescribed antidepressant medication and offered advice relating to diet, exercise, and alcohol consumption compared with patients in other countries (20, 21).

The improved functioning observed in this US cohort of patients with MDD treated with vortioxetine was consistent with previous observations in other real-world populations, with the 8.2-point reduction in SDS total score presented here as comparable to the 8.6- and 8.7-point reductions reported in the RELIEVE Global and RELIEVE China studies, respectively (22, 26). This suggests that the efficacy of vortioxetine remained consistent in the US cohort compared with the RELIEVE global study population despite differences in patient characteristics. This is in contrast to a meta-analysis that suggested that the efficacy of duloxetine versus placebo in clinical trials was lower in countries with higher per capita income and per capita healthcare expenditure, such as the United States (23, 24).

Furthermore, several other studies have demonstrated significant improvements in cognitive symptoms (7, 15, 16) and overall function (27) among patients with MDD treated with vortioxetine. Improved cognition also appears to be a direct effect of vortioxetine, independent of any improvement in depression severity, via a mechanism that is incompletely understood (15, 28, 29).

In the present study of patients in the US cohort, clinically meaningful functional improvements at work/school, in social situations, and with family/home responsibilities were sustained at week 24, along with improvement in depression severity. Improved cognitive symptoms were also observed, and this finding is of particular interest given that cognitive impairment is often a residual feature of MDD despite antidepressant therapy (6, 11, 12).

The results observed in this cohort are also consistent with randomized controlled trials and meta-analyses in which vortioxetine was found to offer significant benefits in improving cognitive symptoms in patients with MDD (15, 16, 30, 31). In the meta-analyses, SNRIs were the only other class of antidepressant that was found to potentially improve cognitive symptoms, although the improvement in cognitive symptoms with SNRI treatment was non-significant compared with placebo (32, 33). In contrast, a non-significant worsening of cognitive symptoms was observed with SSRIs and monoamine oxidase inhibitors in a meta-analysis, and a significant worsening versus placebo was seen with tricyclic antidepressant therapy (30).

The low incidence of treatment-related sexual dysfunction in patients treated with vortioxetine is also consistent with earlier observations in randomized clinical trials (17, 18). Further supporting this finding were the minimal changes observed in ASEX scores, with overall improvements noted during the 6 months of treatment. In addition, vortioxetine was well tolerated, with safety findings consistent with the established tolerability profile (32). A small proportion of patients discontinued vortioxetine because of treatment-related AEs, with nausea being the most common AE reported, which is known to generally be dose dependent, often resolving within weeks of initiating vortioxetine treatment (32).

### Limitations

This study is limited by the potential for bias and confounding that can occur in observational studies (33) and the lack of a comparator arm. The heavily pretreated study population with a long duration of current MDE is also a subset of the overall patient population with MDD in the United States, thus limiting the generalizability of these results outside a second- or later-line setting. However, despite this limitation and the geographical restriction of this analysis, observed outcomes remained similar to the global findings (22). The findings in this study are also strengthened by the long-term 6-month follow-up period and naturalistic study design within a heterogeneous real-world population.

## Conclusion

This study provides real-world evidence supporting the effectiveness of vortioxetine for treating patients with MDD in routine clinical practice in the United States. The study demonstrated improvements in functioning, depressive symptoms, and cognitive function in patients with MDD treated with vortioxetine. Outcomes were consistent with the efficacy and safety profile of vortioxetine in randomized controlled trials.

## Data availability statement

The raw data supporting the conclusions of this article will be made available by the authors, without undue reservation.

## Ethics statement

The studies involving human participants were reviewed and approved by Ethical Committees at each participating site. The patients/participants provided their written informed consent to participate in this study.

## Author contributions

EB and KS were involved with the conceptualization and methodology development. HR coordinated, planned, and executed the research activity. GM was a study investigator and responsible for data collection. GM, HR, and KS were responsible for supervision of the study. KS was responsible for formal data analysis, curation, and visualization. DFL, GM, HR, KS, and LC were responsible for data analysis, validation, and interpretation. DFL, EB, GM, HR, KS, and LC participated in the drafting and critical revision of the manuscript. All authors gave final approval of the version to be published; have agreed on the journal to which the article has been submitted; and agree to be accountable for all aspects of the work.
